# Low-level image statistics in natural scenes influence perceptual decision-making

**DOI:** 10.1038/s41598-020-67661-8

**Published:** 2020-06-29

**Authors:** Noor Seijdel, Sara Jahfari, Iris I. A. Groen, H. Steven Scholte

**Affiliations:** 1 Department of Psychology, University of Amsterdam, Amsterdam, The Netherlands; 2 Amsterdam Brain and Cognition (ABC) Center, University of Amsterdam, Amsterdam, The Netherlands; 3 Spinoza Centre for Neuroimaging, Royal Netherlands Academy of Arts and Sciences (KNAW), Amsterdam, The Netherlands; 4 Department of Psychology, New York University, New York, USA

**Keywords:** Computational models, Object vision, Human behaviour

## Abstract

A fundamental component of interacting with our environment is gathering and interpretation of sensory information. When investigating how perceptual information influences decision-making, most researchers have relied on manipulated or unnatural information as perceptual input, resulting in findings that may not generalize to real-world scenes. Unlike simplified, artificial stimuli, real-world scenes contain low-level regularities that are informative about the structural complexity, which the brain could exploit. In this study, participants performed an animal detection task on low, medium or high complexity scenes as determined by two biologically plausible natural scene statistics, contrast energy (CE) or spatial coherence (SC). In experiment 1, stimuli were sampled such that CE and SC both influenced scene complexity. Diffusion modelling showed that the speed of information processing was affected by low-level scene complexity. Experiment 2a/b refined these observations by showing how isolated manipulation of SC resulted in weaker but comparable effects, with an additional change in response boundary, whereas manipulation of only CE had no effect. Overall, performance was best for scenes with intermediate complexity. Our systematic definition quantifies how natural scene complexity interacts with decision-making. We speculate that CE and SC serve as an indication to adjust perceptual decision-making based on the complexity of the input.

## Introduction

During decision-making, observers extract meaningful information from the sensory environment in a limited amount of time. In recent computational accounts of perceptual decision-making, sensory evidence for a decision option is integrated and accumulates over time until it reaches a certain boundary^1,2^. Across these computational accounts, the speed of evidence accumulation is thought to depend on the quality or strength of sensory information available (the drift rate, as formalized with the well-known drift diffusion model^3^).

In the current study, we aimed to investigate how decision-making processes are influenced by low-level image properties, diagnostic of scene complexity. While multiple studies have shown that specific image properties (such as spatial frequency, or stimulus strength) interact with decision-making, they manipulate visual information into “unnatural” stimuli. For example, we recently showed that image quality modulates response inhibition, and decision-making processes^4^, by manipulating the spatial frequencies of images. Ultimately, however, our goal is to understand how decision processes are influenced by information in *natural scenes*^5^.

The scenes that we encounter in our everyday environment do not contain randomly sampled pixels, but adhere to specific low-level regularities called *natural scene statistics*. Natural scene statistics have been demonstrated to carry diagnostic information about the visual environment: for example, slopes of spatial frequency spectra estimated across different spatial scales and orientations ('spectral signatures') are informative of scene category and spatial layout^6–8^. Similarly, the width and shape of histograms of local edge information estimated using single- and multi-scale non-oriented contrast filters have been shown to systematically differ with scene category and complexity^9–11^.

**Figure 1:**
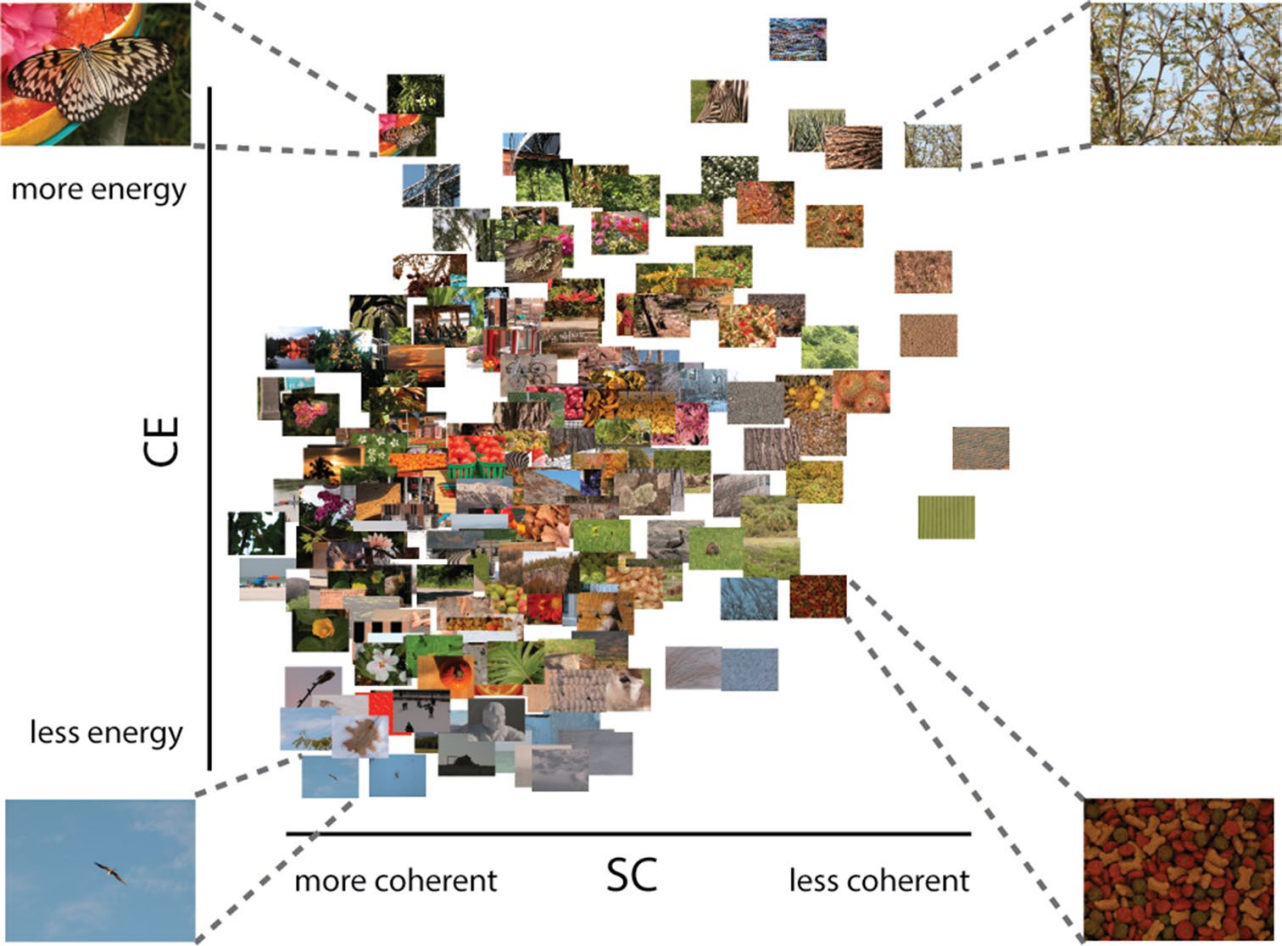
200 real-world scenes plotted against their CE and SC values. Figure adapted from Groen et al. CE and SC reflect different aspects of the local contrast distribution: CE approximates the scale parameter of the Weibull fit and reflects the average local contrast strength in an image. SC approximates the shape parameter of the Weibull fit and reflects to what degree the contrast distribution resembles a power law or Gaussian distribution. Cluttered or complex scenes, with high CE/SC values, have more Gaussian (bell-shaped) distributions compared to sparse or simple scenes with low CE/SC values (power-law shaped), that often contain one or a few salient objects. Four representative pictures are shown in each corner of the parameter space. Scenes that are highly structured (e.g., a street scene) are found on the left, whereas highly cluttered scenes (e.g., a forest) are on the right. Scenes with higher figure-ground segregation (depth) are located on the top, whereas flat images are found at the bottom. Images are from the McGill Calibrated Colour Image Database^18^.

Earlier studies have shown that visual activity evoked by natural scenes can be well described by scene complexity, suggesting that the brain is adapted or tuned to those statistical regularities^10,11^, and potentially using them during visual perception. Scene complexity reflected in local contrast distributions can be estimated using an early visual receptive field model that outputs two parameters, contrast energy (CE) and spatial coherence (SC), approximating the scale and shape of a Weibull fit to the local contrast distribution, respectively (see Supplementary Sect. 1). CE and SC reflect different aspects of the local contrast distribution: CE approximates the scale parameter of the Weibull fit and reflects the average local contrast strength in an image. SC approximates the shape parameter of the Weibull fit and reflects to what degree the contrast distribution resembles a power law or Gaussian distribution. Cluttered or complex scenes, with high CE/SC values, have more Gaussian (bell-shaped) distributions compared to sparse or simple scenes with low CE/SC values (power-law shaped), that often contain one or a few salient objects (Fig. 1; adapted from Groen et al.^12^).

Importantly, CE and SC are computed using a simple visual model that simulates neuronal responses in one of the earliest stages of visual processing. Specifically, they are derived by averaging the simulated population response of LGN-like contrast filters across the visual scene^10^. Similar to other models of representation in early vision (e.g. Ref.^13^), these two-parameters thus provide a compressed representation of a scene. In turn, they could serve as a complexity index that affects subsequent computations towards a task-relevant visual representation.

Here, we investigated whether task-irrelevant manipulations of SC and CE interact with perceptual decision-making by using the drift–diffusion model (DDM)*.* By considering response time distributions for both correct and incorrect choices, the DDM models the speed of evidence accumulation, as well as the amount of evidence required to make a decision. In experiment 1, stimuli were selected such that both CE and SC co-varied with scene complexity, with increasing values representing more complex natural scenes. This is the 'natural situation', since SC and CE are typically correlated within our natural environment. To refine the observations in experiment 1, in experiment 2a and 2b, we also selected stimuli in such a way that the effects for both parameters could be evaluated separately.

## Experiment 1

In experiment 1, we investigated the combined influence of SC and CE on decision-making. As SC and CE are generally highly correlated, varying them together provides the strongest manipulation of information. We expected the drift rate to decrease with increased scene complexity, with an additional shift in the amount of evidence required (boundary) reflecting potential strategic adjustments to the complexity of the scene.

### Methods

#### Participants

Twenty participants (7 males) aged between 18 and 25 years (*M* = 21.9, SD = 1.9) with normal or corrected-to-normal vision, gave written informed consent prior to participation and were rewarded with research credits or monetary compensation. The ethics committee of the University of Amsterdam approved the experiment. All experimental protocols and methods described below were carried out in accordance with the guidelines and regulations of the University of Amsterdam.

#### Stimuli

**Figure 2:**
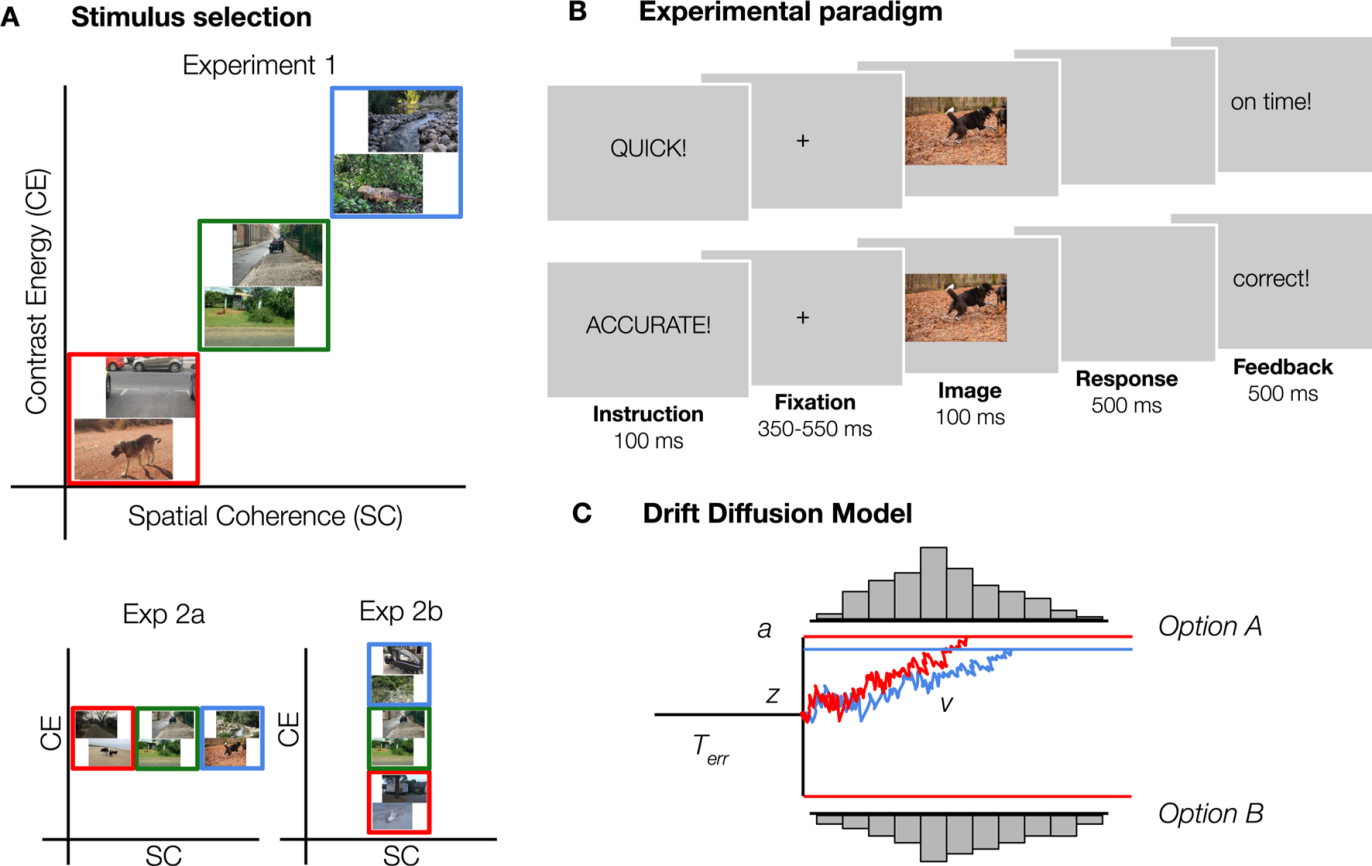
Experimental design and methods. (**A**) Examples of the type of stimuli used in experiment 1, 2a and 2b. Examples shown here were not part of the actual stimulus set. Images varied both in SC and CE (red = low, green = medium, blue = high) in experiment 1. To investigate whether it is meaningful to differentiate between SC and CE, the two parameters were manipulated separately in experiments 2a (SC) and 2b (CE). For each condition, 80 animal and non-animal scenes were selected. (**B**) Experimental paradigm. Participants categorized scenes based on the presence or absence of an animal. On half of the trials, participants were asked to respond as quickly as possible (“speed trials”), as indicated by a pre-cue. On the other half of the trials, participants had to respond as accurate as possible (“accurate trials”). (**C**) Schematic representation of the Drift Diffusion Model. From a starting point *z*, information begins to accumulate in favor of one of the options with drift rate *v* until it reaches a boundary *a*, and the decision is made. Non-decision time *Terr* captures the processes that are unrelated to decision-making, such as response execution.

480 images (640 × 480 pixels, full-color) were obtained from a previous study by Groen et al.^14^. The complete image set contained 7,200 scenes from online databases, including the INRIA holiday database^15^, the GRAZ database^16^, ImageNet^17^ and the McGill Calibrated Color Image Database^18^. For each scene, we computed CE and SC values using the model described in Ghebreab et al. and Groen et al.^11,19^, and selectively sampled scenes for three conditions: low, medium and high (Fig. 2A). Each condition contained 160 scenes, half of which contained an animal. Importantly, within conditions, animal and non-animal scenes were matched in CE and SC values such that these two categories did not differ from each other in mean or median values [mean: all t(158) < 1.14, all *p* > 0.26, median: all z < 1.08, all *p* > 0.28].

#### Procedure

Participants performed an animal/non-animal categorization task^20^ (Fig. 2B). Scenes were presented in randomized sequence, for a duration of 100 ms. Between trials, a fixation-cross was presented with a semi-random duration (350, 400, 450, 500 or 550 ms), averaging to 450 ms. There were two trial instructions, that appeared on screen before every trial in randomly alternating blocks of 20 trials: on “speed trials”, participants were asked to respond as fast as possible, whereas on “accuracy trials”, they responded as accurately as they could. While instruction influences both the accuracy and duration of decisions, the ease of evidence accumulation (drift rate) should remain constant^21^. Using a Speed-Accuracy manipulation allows for a stronger and more sensitive test of the influence of scene complexity on perceptual decision-making. If animal detection in more complex scenes is indeed associated with more cautious or elaborate processing, performance in the high condition should be most affected for “speed trials, in which extensive visual processing is potentially limited by time constraints. Therefore, we aimed to specify how the processing of natural scenes can modulate decision-making processes when participants emphasize accuracy—and allow ample time for processing—or speed. Every scene was presented once for both instructions (960 trials in total). Keyboard buttons were switched halfway (based on a simultaneous EEG study). Comparing % errors in blocks before and after the switch did not indicate switch costs: *M*_*before*_ = *0.13, SD* = *0.15; M*_*after*_ = *0.16, SD* = *0.11*, only taking participants into account for which the same instruction was repeated before and after the switch, averaged across experiments. On speed trials, participants received feedback on their response time ("on time"<500 ms>"too slow"). On accuracy trials, participants were presented with “correct” and “incorrect” feedback. When participants didn't respond, “miss” appeared on screen. Participants were seated ~ 90 cm from the monitor such that stimuli subtended ~ 10 × 14° of visual angle. Images were presented at eye-level on a 23-inch Asus LCD display (sRGB, 2.27 gamma, 1.31 dE) with a spatial resolution of 1,080 × 1920 pixels, at a refresh rate of 60 Hz, using Presentation (version 17.0, Neurobehavioral Systems, Inc.). The ambient illumination in the room was kept constant across different participants.

#### Hierarchical drift diffusion model

We fitted a hierarchical version of the DDM (HDDM^22^) using the RT distributions of correct and incorrect responses. HDDM uses a hierarchical Bayesian estimation, that uses MCMC sampling to estimate the joint posterior distribution of all model parameters, and has been described as method of preference in estimating drift rates for a small number of observations (in the order of 100–20^23^). HDDM assumes that during decision-making, information begins to accumulate from a starting point *z,* in favor of one of the options with drift rate *v* until it reaches a boundary *a*, and the decision is made. Non-decision time *Terr* captures the processes that are unrelated to the decision-making, such as response execution (Fig. 2C).

First, we evaluated five models in which *drift rate* (*v*) and *boundary* (*a*) were either fixed or varied across trial type (speed, accurate) and/or scene complexity (low, medium, high). Using the Deviance Information Criterion (DIC) for model selection we established that, next to varying response boundary across trial type (ΔDIC =  − 3,404 compared to fixed), varying both parameters across scene complexity was justified to account for the data^24^. This fit produced lower DIC values compared to a fit in which the drift rate (ΔDIC =  − 133.3), response boundary (ΔDIC =  − 40.4) or both (ΔDIC =  − 68.1) were fixed across complexity. Then, to assess the trial-by-trial relationship between scene complexity and *drift rate (v)* and *boundary separation (a)*, we fitted eighteen alternative regression models. Both linear models (SC/CE centered around zero), and second-order polynomial models (quadratic) were fitted to examine whether the relationship was curvilinear (e.g. followed an inverted U-shape). We never included both scene statistics simultaneously, as their high correlation leads to multicollinearity and unstable coefficient estimates. To take into account the effect of task instruction on the response boundary *a*, we estimated two intercepts for this parameter (speed and accuracy) using the *depends_on* key argument. For each model, we ran four separate chains with 5,000 samples. The first 200 samples were discarded (burn), resulting in a trace of 19,200 samples. Models were tested for convergence using visual inspection of the group level chains and the Gelman-Rubin statistic, which compares the intra-chain variance of the model to the intra-chain variance of the different runs. It was checked that all group-level parameters had an Rhat between 0.98 and 1.02. For the best fitting model (lowest DIC), we ran posterior predictive checks by averaging 500 simulations generated from the model’s posterior to confirm it could reliably reproduce patterns in the observed data. Bayesian hypothesis testing was performed on the group-level posterior densities for means of parameters. The probability measure P was obtained by calculating the percentage of the posterior < 0 (see Supplementary Sect. 2).

### Results

Data from one participant were excluded for excessive errors (> 23%, 2.8 SD > mean). RTs < 100 ms were considered “fast guesses” and removed. The repeated-measures ANOVA on RT (on correct trials) revealed main effects of both instruction (speed, accurate) and scene complexity (low, medium, high), but no interaction effect, F(36) = 0.261, *p* > 0.77. Similarly, the repeated-measures ANOVA on error rates revealed main effects but no interaction effect, F(36) = 0.177, *p* > 0.83. As expected, responses were faster and less accurate when given a “speed” instruction, in comparison to “accurate”. Because there was no interaction, RTs and error rates were collapsed over speed and accurate trials to further understand how scene complexity modulates decision-making. Bonferroni correction was used for all comparisons.

**Figure 3:**
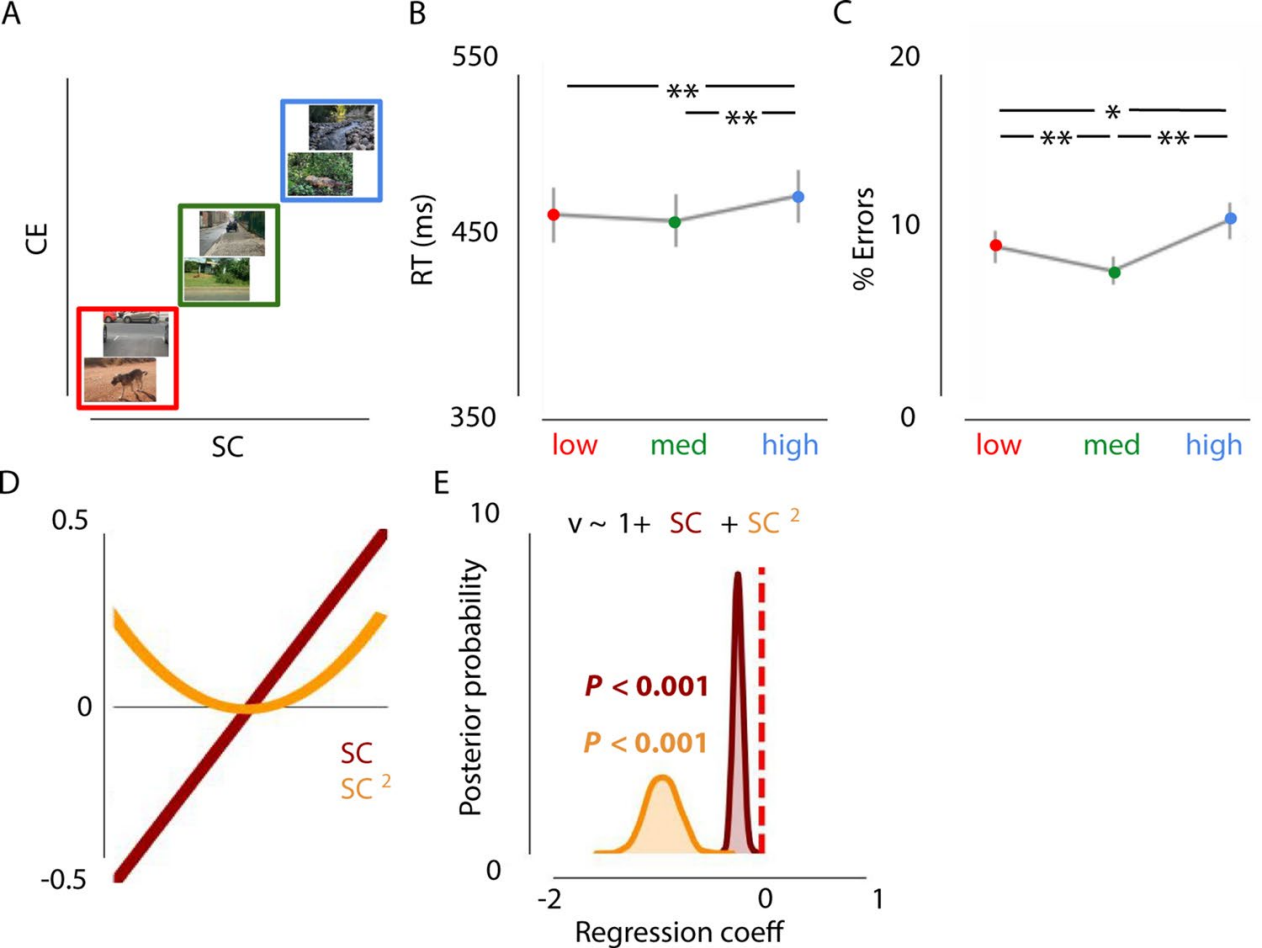
Effects of Spatial Coherence and Contrast Energy on animal vs. non-animal categorization. (**A**) Examples of the type of stimuli used in experiment 1 (not part of actual stimulus set). Images varied both in SC and CE (red = low, green = medium, blue = high). (**B, C**) Results of experiment 1 indicate worse performance for images with high SC/CE, as indicated by higher RTs and lowered accuracy. Error bars represent 1 SEM. *p < .05, **p < .01, Bonferroni corrected. Task performance was best for medium SC/CE images. (**D**) Schematic representation of the linear and quadratic terms included in the regression model. (**E**) Low or high complexity (SC, strongly correlated to CE) was associated with a lower rate of evidence accumulation.

A repeated-measures ANOVA, with factor scene complexity differentiated RTs across the three conditions, F(2,36) = 19.81, *p* < 0.001, η^2^par = 0.524 (Fig. 3A,B). Participants responded slower for high (complex) scenes than for medium-, t(18) = − 7.293, *p* < 0.001, and low scenes, t(18) =  − 3.914, *p* = 0.001. There was also a main effect on error rates, F(2, 36) = 14.26, *p* < 0.001, η^2^par = 0.442. Participants made more errors for high scenes than for medium, t(18) =  − 4.493, *p* < 0.001, and low scenes, t(18) =  − 2.752, *p* = 0.013. Remarkably, participants made fewer mistakes on medium scenes than on low SC/CE scenes, t(18) = 3.405, *p* = 0.003 (Fig. 3C).

Thus, based on the RTs and error rates, we were able to observe a decrease in performance for low and high complexity scenes. To understand this decrease in performance, we modeled the decision variables drift rate (speed of evidence accumulation) and response boundary (evidence requirements). Relative to the null model, the model in which only drift rate was affected by both SC and SC^2^ provided the best fit (ΔDIC = − 71.0, Fig. 3D), compared to models only including the centered or squared SC values and/or including a varying response boundary (see Supplementary Sect. 2). That is, low and high SC were associated with a decreased drift rate (inverted U-shape; *P* < 0.001, Fig. 3E), as indicated by a negative shift in the posterior distribution. In other words, scene complexity influenced the speed of information accumulation, resulting in higher reaction times and more errors for low and high complexity scenes.

## Experiment 2

A key question is whether the effects found in experiment 1 are driven by the two scene statistics together, as they are generally highly correlated in our natural environment, or whether one of them is the primary cause, as suggested by the SC preference in our optimal HDDM model. To refine our interpretation, we systematically manipulated SC while keeping CE constant (experiment 2a) and vice versa (experiment 2b). Experimental procedure and analyses occurred as in experiment 1, except where otherwise indicated.

### Methods

#### Participants

Twenty-four participants (4 males; aged 18–28 years, *M* = 21.8, *SD* = 2.7) participated in experiment 2a; twenty-seven participants (7 males; aged 18–27 years, *M* = 21.4, *SD* = 2.5) participated in experiment 2b. All participants gave written informed consent prior to participation and were rewarded with research credits or monetary compensation. The ethics committee of the University of Amsterdam approved the experiment, and all experimental protocols and methods described below were carried out in accordance with the guidelines and regulations.

#### Stimuli

A new selection of 480 scenes was composed from the same image set as in experiment 1, except that each condition was now defined by either its SC (experiment 2a) or its CE (experiment 2b) values while the other was kept constant at intermediate values (Fig. 2A).

#### Hierarchical drift diffusion model

In experiment 2a we established that, next to varying response boundary across trial type (ΔDIC =  − 3,426 compared to fixed), varying both parameters across SC was justified to account for the data. This fit produced lower DIC values compared to a fit in which the drift rate (ΔDIC =  − 70.5), response boundary (ΔDIC =  − 60.3) or both (ΔDIC =  − 27.8) were fixed across complexity. Next, we evaluated nine regression models to assess the trial-by-trial relationship between scene complexity (indexed solely by SC), and drift rate and response boundary. For experiment 2b model selection indicated that a model in which, apart from varying response boundary across trial type, the parameters were fixed across CE best explained the observed data. This fit produced lower DIC values compared to a fit in which the drift rate (ΔDIC =  − 44.7), response boundary (ΔDIC =  − 76.7) or both (ΔDIC =  − 48.5) were allowed to vary across complexity. Thus, variability in CE alone seems to have no influence on the speed of evidence accumulation or the amount of information required to make a decision. As such, further regression analyses were not justified.

### Results experiment 2a

One participant did not complete the experiment and was excluded from analyses. In contrast to experiment 1, the repeated-measures ANOVA on error rates and RTs showed, apart from the main effects of instruction and scene complexity, an interaction effect, F(42) = 4.351, *p* = 0.0189. Therefore, behavioral results were analyzed separately for “speed” and “accurate” trials to further understand how SC differentially impacts fast or accurate decision strategies.

**Figure 4:**
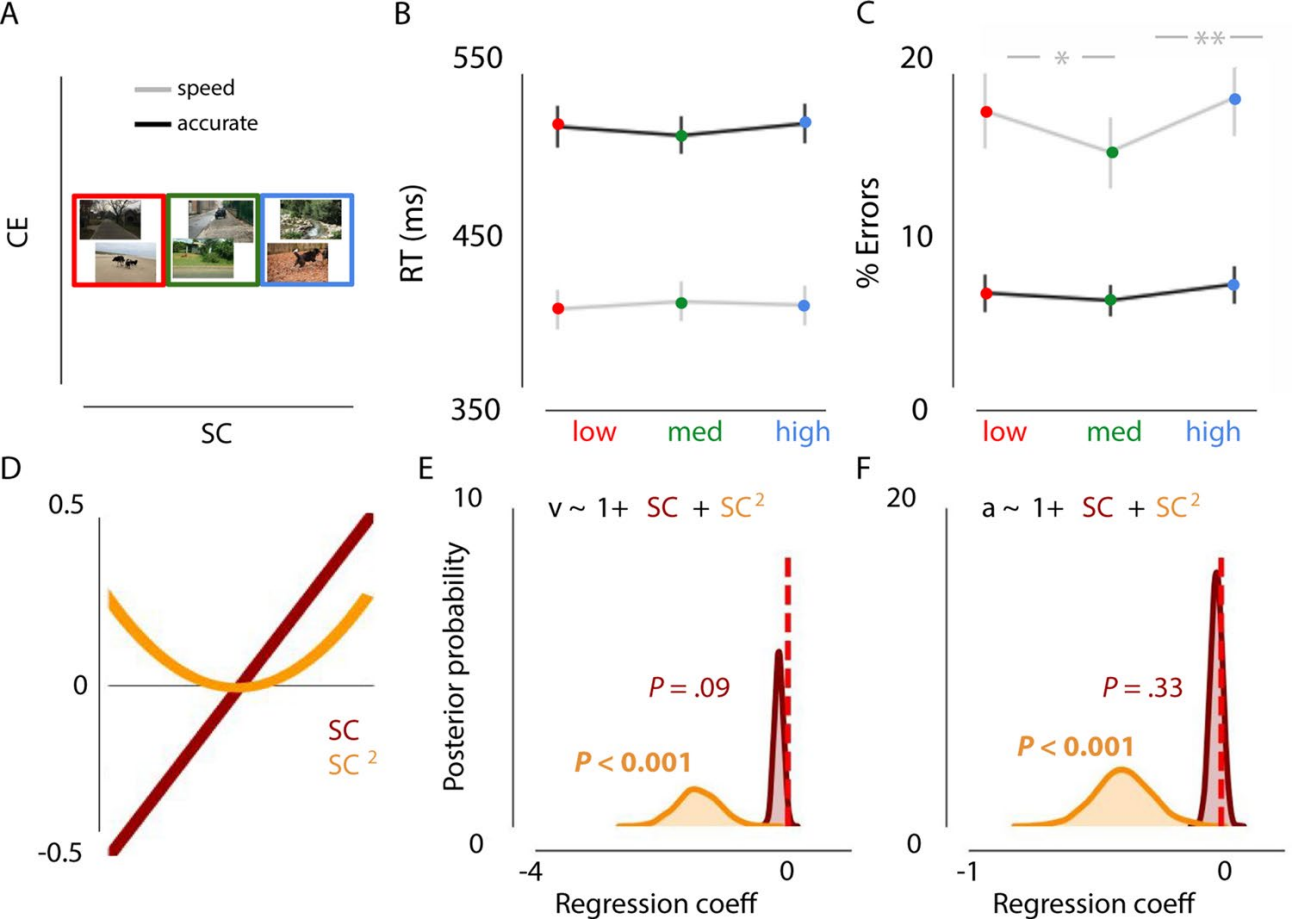
Effects of SC (controlling for contrast energy) on decision-making. (**A**) Examples of the type of stimuli used in experiment 2a (not part of actual stimulus set). Images only varied in SC, while CE was kept constant (red = low SC, green = medium SC, blue = high). (**B**) Results showed no influence of SC on RT. (**C**) Performance was most optimal for images with medium SC complexity in the speed condition, as indicated by a higher accuracy. Error bars represent 1 SEM. *p < .05, **p < .01, Bonferroni corrected. (**D**) Schematic representation of the linear and quadratic term included in the regression model. (**E, F**) Negative shifts in the posterior distributions indicated that low or high complexity (SC) was associated with a lower rate of evidence accumulation and required less evidence to reach a decision (inverted U-shape).

The repeated-measures ANOVA revealed no main effect of SC on RTs for speeded or accurate trials (Fig. 4A,B; all *p* > 0.104). For error rates, there was a main effect of SC on speed trials, F(1, 44) = 9.189, *p* < 0.001, η^2^par = 0.295. Participants made fewer errors for medium SC scenes compared to both low, t(22) = 3.294, *p* = 0.003 or high, t(22) = − 4.346, *p* < 0.001 (Fig. 4C). Notably, SC had no effect on choice errors when participants were motivated to be accurate (*p* > 0.103).

Relative to the null model, the model in which drift rate and response boundary were affected by SC + SC^2^ provided the best fit (ΔDIC = − 21; Fig. 4D). As in experiment 1, low and high SC were associated with a decreased drift rate (inverted U-shape), as indicated by negative shifts in the posterior distribution (*P* < 0.001). Additionally, those scenes were associated with a decreased response boundary (*P* < 0.001; Fig. 4E,F), potentially to still allow for a timely response. Thus, when stimulus information was processed slowly, participants decreased their boundaries, and required less information to reach a decision. When pressed for time, this resulted in more errors for low and high complexity scenes.

### Results experiment 2b

**Figure 5:**
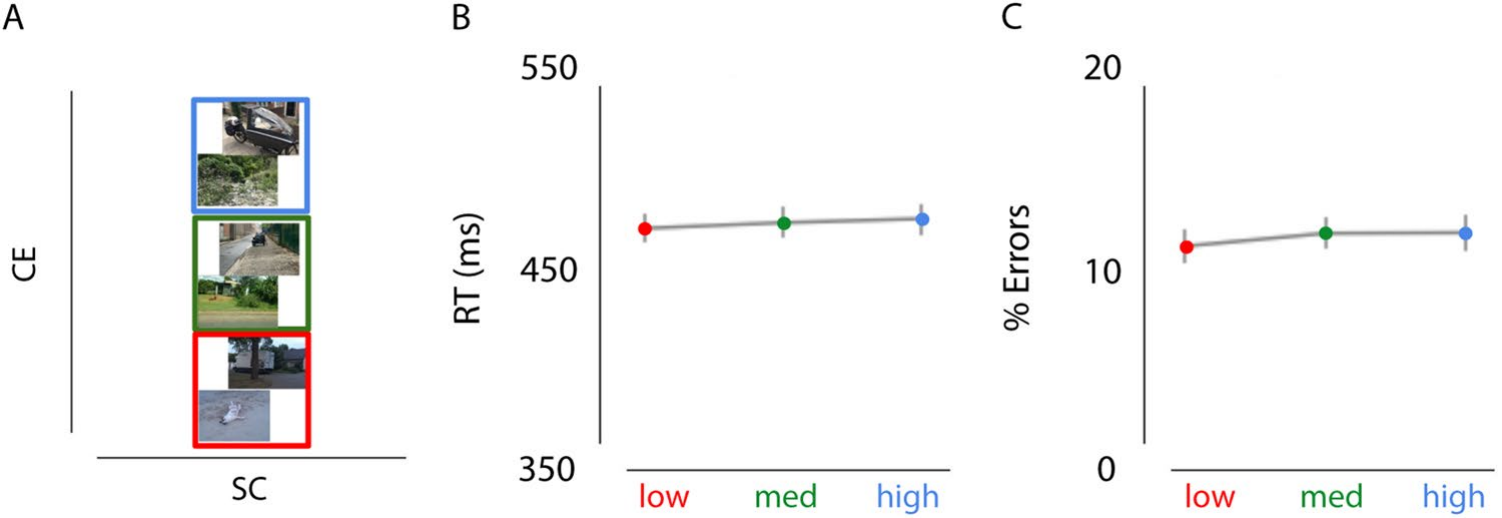
Effects of CE (controlling for spatial coherence) on decision-making. (**A**) Examples of the type of stimuli used in experiment 2b (not part of actual stimulus set). Images only varied in CE, while SC was kept constant (red = low CE, green = medium CE, blue = high CE). (**B, C**) Results of experiment 2b showed no influence of CE on RT or percentage or errors.

Two participants were excluded because of excessive errors (> 25%) or excessive omissions (> 40%). A repeated-measures ANOVA with factors scene complexity (low, medium, high) and instruction (speed, accurate) revealed no interaction effects for RTs, F(48) = 0.093, *p* > 0.9, or errors, F(48) = 1.216, *p* > 0.3. Consistently, no main effect of CE was observed on RTs or errors when speeded and accurate trials were collapsed (Fig. 5; all *p* > 0.306).

## Discussion

This study systematically investigated the interaction between low-level statistics in natural scenes and perceptual decision-making processes. Results indicate that scene complexity, as indexed by two parameters (SC, CE), modulates perceptual decisions through the speed of information processing. Experiment 2a/b refined these observations by showing how the isolated manipulation of SC alone results in weaker yet comparable effects, whereas the manipulation of CE has no effect. By using natural stimuli, we show that task performance was best on medium complex images. Overall, these results show that very basic properties of our natural environment influence perceptual decision-making.

SC and CE together provide a compressed representation of scene complexity. While CE captures information about the amount and strength of edges in a scene, SC indexes higher-order correlations between them, giving an indication of the amount of clutter. In earlier work by Scholte et al. and Groen et al.^10,19,25^, differences in CE were shown to mainly influence the early part of the ERP, while SC effects arose later (up to 300 ms). In the current study, influences on perceptual decision-making seem to be mainly driven by SC. In experiment 1, when SC and CE were both manipulated, model selection indicated a better fit when changes in drift rate were related to SC (as compared to CE), and in experiment 2 only effects of SC were found. Still, there seems to be an additional influence of CE. The finding that there is no interaction between trial type and complexity condition in experiment 1 indicates that even for trials in which there is ample time to process the image, scene complexity influences this process. Thus, while participants were faster and more susceptible to making errors when emphasizing speed (compared to accuracy), emphasis on speed or on accuracy did not change the magnitude of the scene complexity effect on both reaction times and errors. We interpret this as showing that the simultaneous manipulation of SC and CE leads to the strongest effects (as compared to experiment 2). In experiment 2a, in which CE was not manipulated, accuracy was decreased for low and high complexity trials only when participants were pressed for time. This suggests that for low and high complexity scenes, visual information processing might be too slow to produce correct responses, especially when participants are motivated to respond quickly and have lower evidence requirements in comparison to accurate instruction trials. In experiment 2a, low and high complexity scenes were, apart from drift rate, also associated with a lowered response boundary. Overall these results suggest that SC is weighed differently when manipulated in isolation. One explanation for the differences between experiment 1 and 2a could be the inherent correlation between the parameters in the real world, as isolating the influence of both parameters separately could have led to an 'unnatural' sub-selection. For this reason, we cannot attribute our results from experiment 2 exclusively to the scene statistics. Whether this is a robust effect should emerge from future research.

From previous studies, using artificial manipulation of stimulus quality, one would expect performance decreases for more complex scenes. For instance, the search slope of reaction times increases with the number of distractors in conjunction search^26^ and degrading stimulus quality (via spatial filtering) reduces the rate of evidence accumulation^27^. Intuitively 'low' SC scenes are easiest: those scenes are sparser and typically contain the most distinct figure-ground segmentation. Surprisingly, our results suggest a more complex pattern. In experiment 1 and 2a, performance was better on 'medium' than on 'low' scenes. Responses to natural scenes are often hard to predict from studies using artificial stimuli^28^ because the scenes do not contain simple isolated patterns. But why would scenes with medium SC/CE be processed more efficiently? We outline a number of possible reasons below.

First, it could be that scenes with medium complexity are most commonly encountered in daily life, and that the visual system has become tuned to the statistical regularities of medium scenes^29,30^, resulting in optimized visual processing. Secondly, it could be the degree to which object context facilitates the recognition process. In natural scenes, objects appearing in a familiar background are detected more accurately and quickly than objects in an unexpected environment^31–33^. Here, most of the 'low' scenes contained little context because the backgrounds were, generally, homogeneous, providing no ‘cues’ about animal presence or absence. For 'high' images, on the other hand, there may have been too much distraction by spatially unorganized clutter, which does not offer useful cues for animal detection. Third, SC and CE could be related to certain object properties, such as animal size or centrality (the location of the animal in the scene). Additional HDDM analyses however indicated that SC contributed to perceptual decision-making independent of object size, whereas object centrality had no effects (Supplementary Figs. S4–S6)

Finally, SC/CE could be used as diagnostic information, serving as a building block towards estimating other relevant properties in a scene (e.g. scene clutter, naturalness). Since SC correlates with naturalness ratings^19^ and, animals are potentially more strongly associated with natural environments, SC could be a diagnostic feature for the animal/non-animal discrimination task. Indeed, post-hoc evaluation of the responses in experiment 1 and 2a indicated a change in bias towards one of the response options (animal or non-animal), depending on the SC value of the scene. However, the pattern of errors, evaluated for animal and non-animal trials separately, was only partly consistent with a naturalness bias (Supplementary Figs. S7 and S8). In the DDM, effects of a response bias can be explained either by changes in starting point (Δ*z*) or by changes in drift rate (Δ*v*)^34^ or the starting point of the drift rate. Additional modelling suggests that a potential response bias was not reflected in a change in the starting point, and the RT patterns for correct and incorrect trials in our dataset were more in line with a drift bias account (see Supplementary Sect. 5). Crouzet and Serre have shown that low-level image properties such as SC and CE can relate to human performance in an animal detection task^35^. When they trained a classifier to distinguish between animal/non-animal images based on the Weibull parameters (β and γ), classification performance was above chance, but relatively poor compared to alternative models which included more complex visual features, including oriented contrast (V1-like features) and combinations of oriented linear filter responses (mid-level and higher level features). Moreover, the least animal-like stimuli corresponded to more complex backgrounds, while our analyses of response bias (see Supplementary Sect. 4) suggest the opposite pattern. This suggests that the relation between SC, naturalness and animal detection is not trivial and can vary with stimulus set or image database. Here, we carefully selected images to capture a broad range of CE and SC values, and ensured that animal presence was balanced within each condition. Therefore we believe that the current study is a more sensitive test of effects of low-level contrast statistics on perceptual discrimination than previous post-hoc assessments.

In conclusion, the current study provides clear evidence that SC and CE influence perceptual decision-making in an animal detection task. We propose that, because SC and CE could be plausibly computed in early stages of visual processing, they could indicate the need for more cautious or elaborate processing by providing the system with a global measure of scene complexity^36^. Future studies should pinpoint whether this effect is based on the computation of SC and CE directly, as a general measure of complexity, or indirectly, as diagnostic information to estimate other task-relevant scene properties. Given that the rate of evidence accumulation depends on the complexity of the scene, this complexity-dependent adaptation could be reflected in the amount of evidence that is considered sufficient for generating a response. This adaptation, or flexible processing, can help to calibrate the decision process to maximize the goal at hand (e.g. to be accurate or quick).

## Supplementary information

Supplementary file 1 (PDF 504 kb)

## Data Availability

Data and code to reproduce the analyses are available at the Open Science Framework (10.17605/OSF.IO/J2AB9) and at https://github.com/noorseijdel/2019_scenestats.
