# Supplementary material for: Low-level image statistics in natural scenes influence perceptual decision-making
Source: Sci Rep. 2020 Jun 29;10:10573. doi: 10.1038/s41598-020-67661-8 (PMC7324621; doi:10.1038/s41598-020-67661-8)
Supplement: Supplementary file 1 — (PDF 504 kb) [file 41598_2020_67661_MOESM1_ESM.pdf]

Supplementary Materials

for

Low-level image statistics in natural scenes influence perceptual decision-making

Noor Seijdel, Sara Jahfari, Iris I.A. Groen, H. Steven Scholte
Correspondence to: n.seijdel@uva.nl

This document includes:

1. Description of the computation of SC and CE.
2. HDDM model comparison and convergence
3. HDDM Regression analyses incorporating contextual factors.
4. Behavioral analysis to evaluate a potential animal/non-animal bias across the complexity conditions (low, med, high).
5. HDDM analyses evaluating response bias effects across the complexity conditions (low, med, high)

1. Computation of SC and CE

The following section describes the main computational steps. The code to run the model on an arbitrary input image is available on https://github.com/irisgroen/LGNstatistics.

1.1 Natural image statistics: local contrast distribution regularities. Natural images exhibit much statistical regularity, one of which is present in the distribution of local contrast values. It has been observed (Simoncelli, 1999; Geusebroek and Smeulders, 2002, 2005) that properties that are inherent to natural images, such as spatial fragmentation (generated by the edges between the objects in the scene) and local correlations (due to edges belonging to objects in the image) results in contrast distributions that range between power law and Gaussian shapes, and therefore conform to a Weibull distribution. This regularity (systematic variation in the contrast distribution of natural images) can therefore be adequately captured by fitting a Weibull function of the following form:

$$p(f) = c\, e^{(\frac{f-\mu}{\beta})^{\gamma}}$$

Where *c* is a normalization constant that transforms the frequency distribution into a probability distribution. The parameter μ, denoting the origin of the contrast distribution, is generally close to zero for natural images. We normalize out this parameter by subtracting the smallest contrast value from the contrast data, leaving two free parameters per image, β (*beta*) and γ (*gamma*), that represent the scale (*beta*) and shape (*gamma*) of the distribution (Geusebroek & Smeulders, 2002, 2005). Beta varies with the range of contrast strengths present in the image, whereas gamma varies with the degree of correlation between contrasts.

1.2 LGN model of local contrast statistics: contrast energy and spatial coherence. In previous work, we found that the beta and gamma parameters of the Weibull distribution can be approximated in a physiologically plausible way by summarizing responses of receptive field models to local contrast (Scholte et al., 2009). Specifically, summing simulated

receptive field responses from a model of the parvocellular and magnocellular pathways in the LGN led to accurate approximations of beta and gamma, respectively. In subsequent papers (Groen et al., 2013, 2017) an improved version of this model was presented in which contrast was computed at multiple spatial scales and the LGN approximations were estimated not via summation but by averaging the local parvocellular responses (for beta) and by averaging and divisively normalizing the magnocellular responses for gamma (mean divided by standard deviation). To distinguish the Weibull fitted parameters from the LGN approximations, the LGN-approximated beta value was defined as Contrast Energy (CE) and the LGN-approximated value of gamma as spatial coherence (SC). These modifications, as well as specific parameter settings in the model, were determined based on comparisons between the Weibull fitted values and the CE/SC values, as well as model fits to EEG responses, in separate, previously published image sets (Ghebreab et al., 2009, Scholte et al., 2009). We outline the main computational steps of the model below:

1.3 main computational steps of the model

Step 1: RGB to color opponent space. For each image, the input RGB values were converted to opponent color space using the Gaussian color model described in (Geusebroek, Van den Boomgaard, Smeulders & Geerts, 2001), yielding 3 opponent color values per pixel (grayscale, blue-yellow, red-green; Koenderink, Van De Grind & Bouman, 1972).

Step 2: Multi-scale local contrast detection. Each color opponent layer was convolved with isotropic exponential filters (Zhu and Mumford, 1997) at five octave scales (Croner and Kaplan, 1995). Two separate filter sets were used: smaller filter sizes (0.12, 0.24, 0.48, 0.96, and 1.92 degrees) for CE and larger filter sizes (0.16, 0.32, 0.64, 1.28, and 2.56 degrees) for SC (Ghebreab et al., 2009). Following the LGN suppressive field approach (Bonin et al., 2005), all filter responses were rectified and divisively normalized.

Step 3: Scale selection. Per parameter (CE or SC) and color-opponent layer, one filter response was selected for each image location from their respective filter set using minimum reliable scale selection (Elder and Zucker, 1998). In this MIN approach, the smallest filter size that yields an above-threshold response is preferred over other filter sizes. Filter-specific noise thresholds were determined from a separate image set (Corel database) (Ghebreab et al., 2009).

Step 4: Spatial pooling. Applying the selected filters for each image location results in two contrast magnitude maps: one highlighting detailed edges (from the set of smaller filter sizes, for CE) and the other more coarse edges (from the set of larger filter sizes, for SC) per color opponent-layer. To simulate the different visual field coverage of parvo- and magnocellular pathways, a different amount of visual space was taken into account for each parameter in the spatial pooling step. For CE, the central 1.5 degrees of the visual field was used, whereas for SC, 5 degrees of visual field was used. Finally, the estimated parameter values were averaged across color-opponent layers resulting in a single CE and SC value per image.

2 HDDM model comparison and convergence

First, we evaluated five models in which drift rate (v) and boundary (a) were either fixed or varied across trial type (speed, accurate) and/or scene complexity (low, medium, high).

Supplementary Table S1. HDDM model fits to determine whether varying across scene complexity was justified to account for the data. We evaluated five models in which drift rate (v) and boundary (a) were either fixed or varied across trial type (speed, accurate) and/or scene complexity (low, medium, high).

	drift rate (*v*)	boundary (*a*)	DIC - exp 1	DIC - exp 2a	DIC - exp 2b
model 0	-	-	-11453.46	-8545.74	-7240.21
model 1	-	instruction	-14857.92	-11971.86	**-10109.50**
model 2	complexity	instruction	-14885.64	-11939.36	-10064.76
model 3		instruction, complexity	-14792.76	-11929.10	-10032.79
model 4	complexity	instruction, complexity	**-14926.04**	**-11999.62**	-10060.99

Then, to assess the trial-by-trial relationship between scene complexity and drift rate (v) and boundary separation (a), we fitted eighteen alternative regression models.

Supplementary Table S2. HDDM Regression models. Drift rate *v* and boundary *a* were either allowed to vary across scene complexity (indexed by SC or CE) or fixed. Both linear models (SC/CE centered around zero), and second-order polynomial models (quadratic) were fitted to examine whether the relationship was curvilinear (e.g. followed an inverted U-shape).

~	drift rate (*v*)	boundary (*a*)	both (*v, a*)
SC	model 1	model 2	model 3
SC^2	model 4	model 5	model 6
$SC+SC^2$	model 7	model 8	model 9
CE	model 10	model 11	model 12
CE^2	model 13	model 14	model 15
$CE+CE^2$	model 16	model 17	model 18

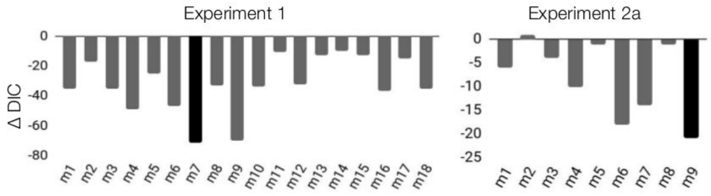

Supplementary Table S3. Means of the posterior distributions.

parameter	Experiment 1	Experiment 2a
t	0.18	0.23
z	0.27	0.29
v_Intercept	3.69	3.16
v_SC	0.23	-0.14
v_SC_squared	-0.96	-1.43
a_Intercept(Ac)	1.93	1.85
a_Intercept(Sp)	1.46	1.47
a_SC	-	-0.02
a_SC_squared	-	-0.39

3. HDDM analyses incorporating contextual factors

The following section describes the methods for the additional analyses to evaluate potential contextual factors that could correlate with SC and limit the detection task. Specifically, we parameterized two characteristics, object size and centrality. We have focused on these two factors, because just like CE and SC, they were image-computable, i.e. they could be derived by performing calculations on the pixels in the image.

3.1 Computing contextual factors

Object size was computed by taking the percentage of the image that was covered by the animal (manually segmented). Object centrality was computed by taking the distance in pixels from the center of mass (CoM) of the animal (computed by interpreting the image as a 2D probability distribution) to the center of the screen (see Supplementary Figure S1).

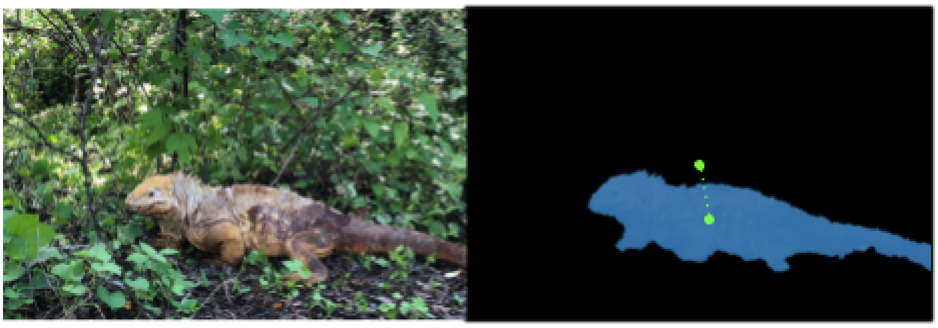

Supplementary Figure S4. Example of computing object (animal) coverage and centrality. Object size was computed by taking the percentage of the image that was covered by the animal (manually segmented). Object centrality was computed by taking the distance from the center of mass (CoM) of the animal to the center of the screen (length of green dotted line, in pixels).

3.2 Evaluating the relationship with SC and CE

There was no correlation between SC or CE and object coverage (experiment 1; SC: r = 0.018; CE: r = 0.025) or centrality (SC: r = -0.13; CE: r = -0.09). To evaluate whether SC explains unique variance after accounting for these properties, we included both variables in our HDDM regression analysis, alongside SC.

For experiment 1, results showed an influence of object size (coverage) on the drift rate, with a higher drift rate for images with larger animals as indicated by a positive shift in the posterior distribution (Supplementary Figure S2; $P < .001$). For object centrality, however, we found no effect, and inspection of this variable indicated a low variability: most animals were located quite central. In experiment 2a, as in experiment 1, larger animals were associated with a higher drift rate (Supplementary Figure S3; $P < .001$).

Most importantly, for both experiments, the effect of SC remained. This indicates that, even though object size has an influence on the rate of evidence accumulation, SC continues to explain unique variance in the speed of information processing. In other words, SC contributes to perceptual decision-making independent of object size, whereas object centrality has no effects.

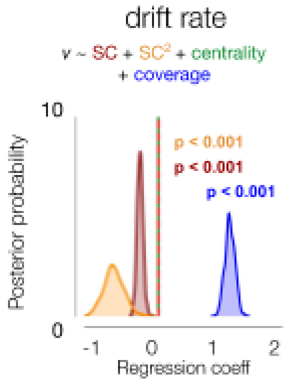

Supplementary Figure S5. Effects of SC/CE (experiment 1) on drift rate, accounting for object size and centrality. ***A/B)*** Bigger animals were associated with a higher rate of evidence accumulation. The effects of SC+SC^2 remained, indicating that, even though object size has an influence on the rate of evidence accumulation, SC continues to explain unique variance in the speed of information processing.

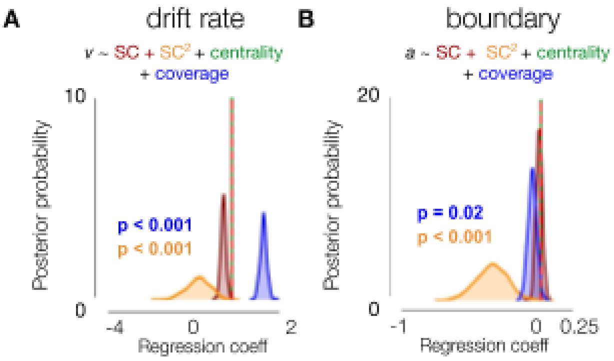

Supplementary Figure S6. Effects of SC (experiment 2a) on drift rate and response boundary, accounting for object size and centrality. A/B) Bigger animals were associated with a higher rate of evidence accumulation. Again, the effect of SC^2 remained, indicating that even though object size has an influence on the rate of evidence accumulation, SC continues to explain unique variance in the speed of information processing.

Full description and code definitions can be found here:
https://github.com/noorseijdel/2019_scenestats/blob/master/notebooks/Notebook_SceneStats_Context.ipynb

4 Behavioral analysis evaluating animal/non-animal bias

To investigate whether participants' response bias (towards animal or non-animal) differed with scene complexity, we computed the % animal choices for each participant. Differences between the three conditions (low, med, high) were statistically evaluated using a repeated-measures ANOVA.

For experiment 1, results indicated, apart from a general bias towards the non-animal option (animal choice < 50% for all conditions), that the % animal-responses increases with scene complexity, $F(36) = 9.76$, $p < 0.001$, $\eta^{2par} = .351$ (Supplementary Figure S4). Participants

chose ‘animal’ more often in the high and medium complexity scenes as compared to low, t(18) = -5.104, p < .001; t(18) = -2.698, p = .044 (Bonferroni corrected). Similar effects were found for experiment 2a (Supplementary Figure S5). There, the percentage of animal responses increased with SC, F(44) = 6.63, p = 0.003, η^{2par} = .232. Participants chose 'animal' more often in the high scenes as compared to low, t(22) = -3.365, p = .008 (Bonferroni corrected).

In the current experiment, half of the trials in each condition contained an animal. Therefore, this response bias towards animal or non-animal trials can result in an increase in errors in the low and high condition. Analysis of the error rates separately for animal and non-animal trials, indicated for both experiment 1 and experiment 2a that most errors in the low condition were made for animal-trials. In those trials, participants thus seem to ‘miss’ the animal more often. Errors in high scenes, however, were seemingly not caused by the response bias: while participants reported more animals on non-animal trials (compared to low and medium), they made as many errors on animal trials.

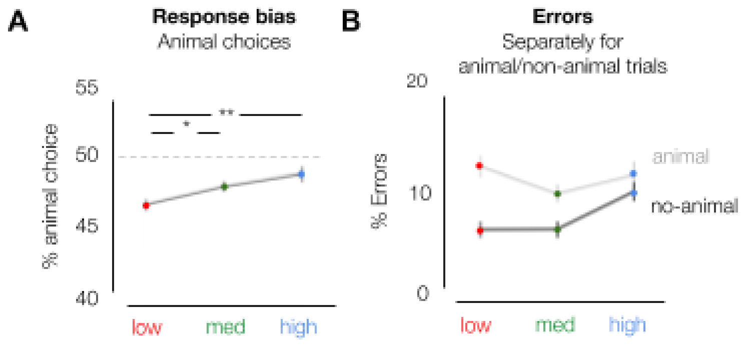

Supplementary Figure S7. Response bias effects in experiment 1. A) apart from a general bias towards the non-animal option (animal choice < 50% for all conditions), the % animal-responses increased with scene complexity. **B)** percentage of errors from experiment 1, separately for animal and non-animal trials.

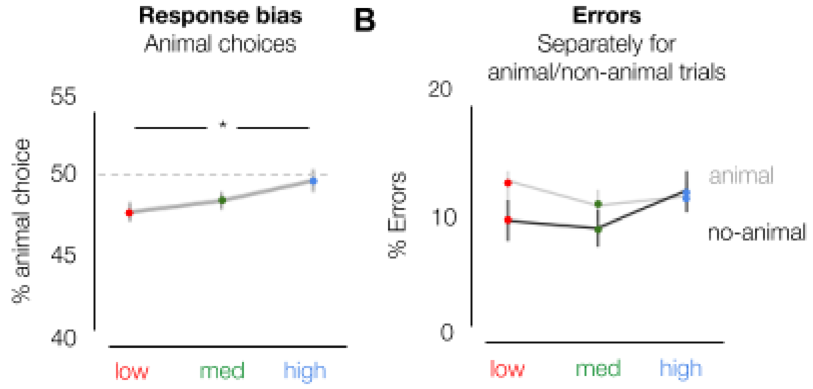

Supplementary Figure S8. Effects of SC on animal/non-animal responses in experiment 2a. A) Similar to experiment 1, the % animal-responses increased with SC. **B)** Percentage of errors from experiment 2a, plotted separately for animal and non-animal trials.

5 HDDM Regression analyses evaluating response bias effects

Following Supplementary section 4, to assess whether SC biases the response (towards animal or non-animal) reflected in changes in the starting point, we fitted several HDDMRegressor models:

1. one model in which we estimate only the response bias z for every complexity condition (low, med, high), such that the bias for animal stimuli is z and the bias for non-animal stimuli is 1-z (z = 0.5 for unbiased decisions in HDDM.
2. one model in which we estimate both v and z. This way, we could measure response-bias (in favor of animal or non-animal) and drift rate for the three conditions (low, med, high) while assuming the same drift rate for both stimuli.
3. one model in which we estimate v, z and a for every complexity condition.
4. one model in which we estimate v, z and a for every complexity condition and, using the depends_on key argument, two intercepts for a (speed, accurate)
5. same model as 4, now using 'medium' as the intercept for z

However, with the properties of our observations and design, models defined in this way do not converge, which makes the interpretation of the parameters uninformative. The traces are non-stationary, and the autocorrelation is high. The histograms look serrated.

Full description and code definitions can be found here:
https://github.com/noorseijdel/2019_scenestats/blob/master/notebooks/Notebook_SceneStats_ResponseBias.ipynb

Then, we fit

6. one model using HDDMStimCoding, in which we estimate v, z for every complexity condition, and a for every complexity condition + speed/accuracy instruction.

This model converges. As shown in the figure (Supplementary figure S9) below, the obtained posteriors for z do not differ across our low, med, or high condition. Hence, this evaluation shows no effect of condition (low, med, high) on z when it is allowed to vary.

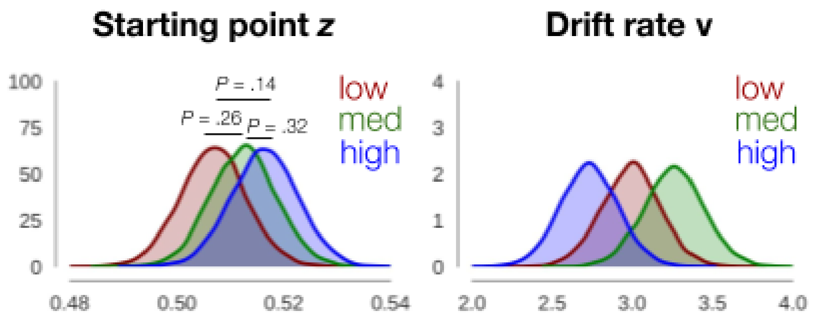

Supplementary Figure S9. Using HDDMStimCoding to evaluate potential biases towards animal/non-animal across the different conditions for the data obtained in experiment 1.

In the DDM, effects of a response bias can be explained either by changes in starting point (Δz) or by changes in drift rate (Δv; Mulder, Wagenmakers, Ratcliff, Boekel & Forstmann, 2012)) or the starting point of the drift rate. Additional modeling suggests that a potential response bias was not reflected in a change in the starting point and the RT patterns for correct and incorrect trials in our dataset were more in line with a drift bias account:

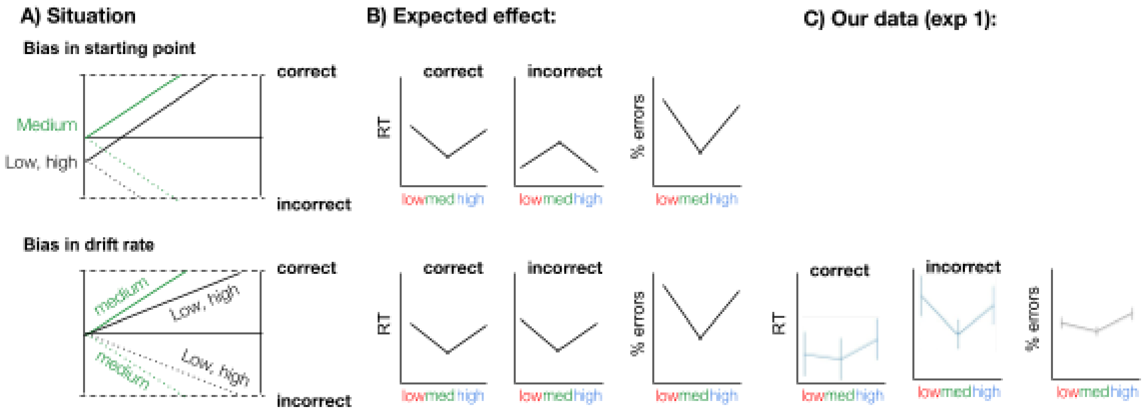

Supplementary Figure S10. Possible effects of bias on choice behavior (following figure 2 from Mulder et al., 2012). A) Effects of bias explained by the drift-diffusion model. When prior information is invalid ('low', 'high') for the choice at hand, subjects will have slower and less correct choices compared with choices where no information is provided (neutral, 'medium'). These effects can be explained by changes in the starting point or the drift rate of the accumulation process. B) Both accounts have different effects on RT and accuracy data. C) The data from our current experiment is more in line with a drift rate account of response bias.
